# The Global Landscape of the Burden of Depressive Symptoms/Major Depression in Individuals Living With HIV/AIDs and Its Effect on Antiretroviral Medication Adherence: An Umbrella Review

**DOI:** 10.3389/fpsyt.2022.814360

**Published:** 2022-05-12

**Authors:** Mogesie Necho, Yosef Zenebe, Chalachew Tiruneh, Getinet Ayano, Bethlehem Yimam

**Affiliations:** ^1^Department of Psychiatry, College of Medicine and Health Sciences, Wollo University, Dessie, Ethiopia; ^2^Department of Anatomy, College of Medicine and Health Sciences, Wollo University, Dessie, Ethiopia; ^3^Research and Training Department, Amanuel Mental Specialized Hospital, Addis Ababa, Ethiopia

**Keywords:** depression, depressive symptoms, HIV/AIDS, review of reviews, global

## Abstract

**Background:**

People living with HIV/AIDS have a higher rate of depression/depressive symptoms and this highly affects antiretroviral medication adherence. Therefore, much stronger evidence weighing the burden of depressive symptoms/major depression is warranted.

**Methods:**

We investigated PubMed, Scopus, Psych-Info, and Embase databases for systematic review studies. A PRISMA flow diagram was used to show the search process. We also used the Assessment of Multiple Systematic Reviews (AMSTAR) checklist scores. A narrative review and statistical pooling were accompanied to compute the pooled effect size of outcome variables.

**Results:**

Overall, 8 systematic review studies addressing 265 primary studies, 4 systematic review studies addressing 48 primary studies, and six systematic review studies addressing 442 primary studies were included for depressive symptoms, major depression, and their effect on medication non-adherence, respectively. Globally, the average depressive symptoms prevalence using the random effect model was 34.17% (24.97, 43.37). In addition, the average prevalence of major depressive disorder was obtained to be 13.42% (10.53, 16.31). All of the 6 included systematic review studies reported a negative association between depressive symptoms and antiretroviral medication non-adherence. The pooled odds ratio of antiretroviral medication adherence among patients with depressive symptoms was 0.54 (0.36, 0.72) (*I*^2^ = 0.0%, *p* = 0.487).

**Conclusion:**

Globally, the prevalence of depressive symptoms and major depression is high. There existed a high degree of association between depressive symptoms and antiretroviral medication non-adherence. So, focused intervention modalities should be developed and implemented.

## Background

Individuals living with HIV/AIDS are more affected by this mental health problem than the general population up to 3 times higher (1). Depressive symptoms and the prevalence of depression are increased in people living with human immune virus (PLWHIV) and the presence of depressive symptoms or depression is associated with decreased adherence to antiretroviral medication.

The global prevalence of depressive symptoms among people living with HIV/AIDS reaches 78% as evidenced from a review study by Uthman et al. (2). A sub-Saharan Africa review study that integrated 30 studies from 10 African countries revealed that the pooled prevalence of depression in patients with HIV/AIDS was 31.2% (3). Another sub-Saharan Africa review study revealed that depression symptom prevalence reaches 32% (4). A review study by Ayano et al. (5) on the east African HIV/AIDS population also obtained a high prevalence of depression (38%).

Major depressive disorder/depressive symptoms are linked to many undesired treatment outcomes in individuals living with HIV/AIDS (6), partially owing to the intermediating part of antiretroviral therapy (ART) non-adherence. It is known that depression is related to non-adherence in numerous long-lasting physical health conditions (7, 8); the probabilities of health non-adherence to medical prescriptions being more than 3 times higher in patients having depressive disorders/symptoms than patients not having depressive disorders (8). A systematic review and meta-analysis study consisted of 95 studies that reported that the presence of depressive disorder meaningfully affected ART medication non-adherence (9).

Results from a longitudinal study implied that reductions in depression symptoms over time were strongly connected with enhancements in ART treatment adherence and better treatment outcome (10). Another systematic review and meta-analysis study consisted of 29 studies and 12,243 participants living with HIV/AIDS reported that the effective treatment for depressive symptoms/disorder and emotional distress enhanced antiretroviral medication adherence and patients' overall treatment outcome (11).

The low level of policy consideration on depression among people living with HIV/AIDS and its effect on the antiretroviral medication adherence as well as the congruently inadequate implementation practice of interventions in this population might be attributed to the inadequacy of well-aggregated data regarding the extent of the problem. The need to develop a systematic review of reviews for a better evaluation, complementary data, and invention of evidence for policymakers and intervention planners is essential. Therefore, this umbrella review would have potential implications for different health sector stakeholders for having conclusive evidence regarding the problem and designing appropriate intervention plans and clinical guidelines.

## Methods

### Definition of an Umbrella Review

An umbrella review also known as a systematic review of systematic reviews is a synthesis that integrates previously done systematic reviews and meta-analysis studies, to provide very strong and concrete evidence in a given area. The evidence obtained from this review is essential for the development of health care policy, treatment, and prevention guidelines (12, 13). As with systematic review and meta-analysis studies, an umbrella review follows a systematic approach in searching the articles, quality appraisal, synthesis, and reporting of the results (14–16).

### Search Strategy and Inclusion Criteria for Systematic Reviews

We searched PubMed, Scopus, Psych-Info, and Embase databases for systematic reviews for available eligible studies. The main objectives of the search for this umbrella review were the burden of depressive symptoms/depressive disorder and its effect on antiretroviral medication non-adherence.

Sample of the search strategy for depressive symptoms/major depressive disorder in PsycINFO *via* Ovid: (((depressive symptoms.mp. [mp = title, abstract, heading word, table of contents, key concepts, original title, tests, & measures]) OR (major depressive disorder.mp. [mp = title, abstract, heading word, table of contents, key concepts, original title, tests, & measures]))) OR (depression.mp. [mp = tile, abstract, heading word, table of contents, key concepts, original title, tests, & measures]))) AND (HIV.mp. [mp = title, abstract, heading word, table of contents, key concepts, original title, tests, & measures]))) OR (AIDS.mp. [mp = tile, abstract, heading word, table of contents, key concepts, original title, tests, & measures]))) OR (acquired immunodeficiency syndrome.mp. [mp = tile, abstract, heading word, table of contents, key concepts, original title, tests, & measures]))) AND (((review.mp. [mp = title, abstract, heading word, table of contents, key concepts, original title, tests, & measures]) OR (meta-analysis.mp. [mp = title, abstract, heading word, table of contents, key concepts, original title, tests, & measures] OR (systematic review.mp. [mp = title, abstract, heading word, table of contents, key concepts, original title, tests, & measures]))). Furthermore, Google scholar, and the reference lists of on hand articles were investigated.

### Outcome Measures and Their Operational Definitions

In this umbrella review, the systematic review studies that reported the following outcomes were incorporated.

#### Depressive Symptoms

In the present umbrella review, depressive symptoms among patients with HIV/AIDS were the first outcome variable. It was defined as the average effect size of depressive symptoms reported by a systematic review study or an estimate of depression obtained with varieties of screening tools in a meta-analysis study. Such measurement tools include but are not limited to the Center for Epidemiologic Studies Depression Scale (CES-D); Depression, Anxiety, and Stress Scale (DASS-21); Hospital Anxiety and Depression Scale (HADS); Patient Health Questionnaire-9 (PHQ-9); and Zung Self-Rating Depression Scale.

#### Depressive Disorder

In the present study, the depressive disorder was defined as the reported pooled estimate of depressive disorder by the included systematic review study or the estimated effect size of depression measured with varieties depression diagnostic tools, such as Composite International Diagnostic Interview Short Form (CIDI-SF), Diagnostic and Statistical Manual of Mental Disorders (DSM), and Mini-International Neuropsychiatric Interview (MINI).

#### Effect of Depression on Antiretroviral Medication Non-adherence

One of our outcome variables is the effect of depression on antiretroviral medication non-adherence, and it was operationalized in the study as a measured effect size in terms of association measures between depression and antiretroviral medication non-adherence.

### Inclusion Criteria

A study was included in the current umbrella review if it fulfills the following criteria [1] it must be titled with systematic review/meta-analysis in its heading [2]; depressive symptoms/major depressive disorder and its effect on antiretroviral medication non-adherence was the main objective [3]; incorporated at least two primary studies that investigated depressive symptoms/major depression and/or its effect on antiretroviral medication non-adherence [4]; the language of publications must be in the English language [5] if pooled effect size was calculated in the primary reviews, the model, the methodology, heterogeneity issues, and publication bias were indicated.

### Exclusion Criteria

We excluded systematic review articles that assessed depression/depressive symptoms and their effect on non-adherence to medication in HIV/AIDS patients at extreme age groups (adolescents and old age groups of patients). In addition, we excluded articles that were mere literature reviews that do not follow a standard guideline and articles published in non-English languages. Moreover, editorials reports, qualitative reviews, and reviews that assessed the outcome in either male or female sex only were excluded from the analysis.

### Risk of Bias and Data Extraction

All systematic review and meta-analysis studies retrieved from the search databases were exported to an Endnote database manager. In the Endnote database manager, all articles imported from the different search databases were checked for duplicates and duplicate articles were then removed. The next step was assessing the titles and abstracts of the remaining articles before reviewing full texts. Systematic Review studies that meet the inclusion criteria after full-text review were then assessed using Assessment of Multiple Systematic Reviews (AMSTAR) checklist scores for their quality. The checklist has-11 components which can be summed and scored from 11. An overall score of ≥8, 4–7, and score ≤ 3 was interpreted as high quality, medium quality, and low-quality studies, respectively. Two authors (MN & YZ) individualistically evaluated the quality of each systematic review study. The internal consistency between the two authors was 96% and agreement on the remaining 4% was touched by discussion. Relevant data from the included systematic review studies were extracted in a table having the following format: Name of author and year of publication; geographic coverage of the systematic review; the investigated databases; assessment tool for depressive symptoms/major depression; number of primary studies; the number of studied participants in the systematic review; main findings of the systematic review; and the AMSTAR score.

### Data Synthesis

We synthesize the data separately for depressive symptoms/ major depressive disorder and its effect on antiretroviral medication non-adherence. A narrative review and statistical pooling (meta-analysis using the random effect model) were accompanied to compute the pooled effect size of depressive symptoms/ major depressive disorder and its effect on antiretroviral medication non-adherence. The presence of heterogeneity was confirmed using the Higgins method, with *I*^2^ statistics value ≥50 implying heterogeneity. The existence of publication bias was checked with a funnel plot and Egger's regression test. The Stata-11 software packages were used to compute the effect size (17).

## Result

### Search

**Figure 1:**
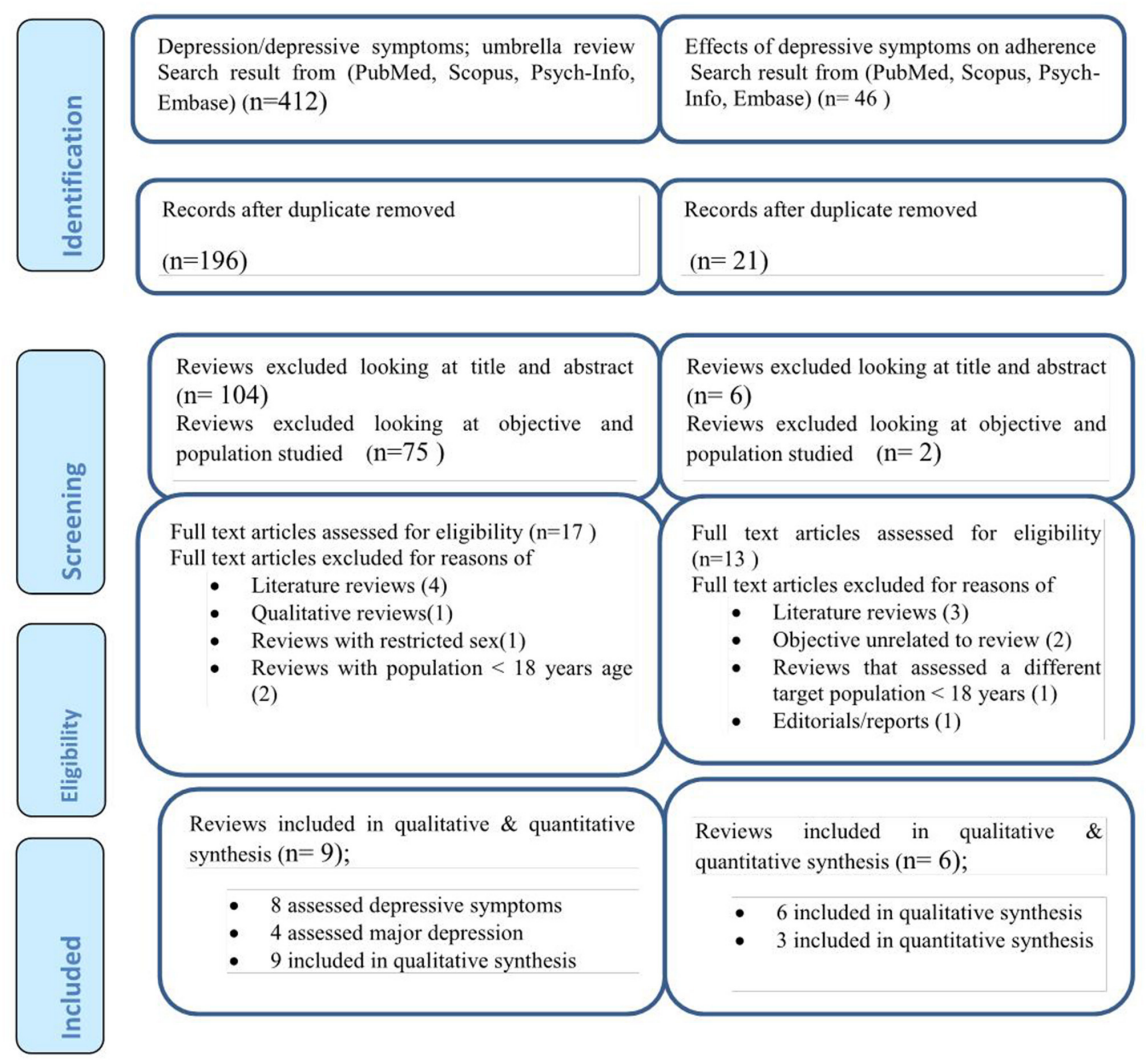
PRISMA flow chart for the review search process.

We searched 412 articles concerning depressive symptoms/major depression in HIV/AIDS patients and 46 articles related to the association between depressive symptoms/major depressions and antiretroviral medication non-adherence. Once duplicate articles were removed and further screening for titles and abstracts were done, 17 systematic reviews for depressive symptoms/major depression prevalence and 13 systematic reviews on the effect of depressive symptoms/major depressions on antiretroviral medication non-adherence were assessed as full-text reviews. Eight systematic reviews studying the depressive symptoms/major depression in HIV/AIDS were excluded due to the following reasons: they were mere literature reviews (2), qualitative reviews (18), reviews addressing only the female sex (18), reviews conducted in HIV/AIDS patients with <18 years ago (19). Similarly, seven of the articles on the effect of depressive symptoms/major depressions on antiretroviral medication non-adherence were excluded as they were literature reviews (1), their objectives are unrelated to the review (19), reviews that assessed a different target population/ <18 years of age (18), and editorials/reports (18). Finally, 8 studies that assessed depressive symptoms (3–5, 20–24), of which 4 studies that assessed major depression (3, 5, 9, 23) were included in the umbrella review for the prevalence of depressive symptoms. Similarly, six studies (2, 3, 9, 11, 25, 26) that assessed the effect of depressive symptoms/depression on antiretroviral medication non-adherence were included in the final analysis (Figure 1).

### Depressive Symptoms/Major Depression Prevalence

#### Characteristics of Included Reviews in the Umbrella Review

Overall, eight systematic review studies (3–5, 20–24) addressing 265 primary studies assessed depressive symptoms and four systematic review studies (3, 5, 9, 23) addressing 48 primary studies that assessed major depression were included in this umbrella review.

Considering the geographical coverage of the review, three reviews cover primary studies from sub-Saharan Africa (3, 4, 23) and two reviews cover primary studies from Ethiopia (20, 21). The number of original studies incorporated in the systematic reviews vary from as low as 10 studies with 2,596 participants (9) to as high as 74 studies with 20,635 participants (24). Patient health questionnaire-9(PHQ-9) was the most predominantly used tool to screen depressive symptoms in four of the review studies (5, 20, 22, 23). PubMed/MEDLINE, Psych INFO, Scopus, and EMBASE databases were the most frequently searched databases by the systematic review studies. Only six systematic review studies assessed the quality of primary studies using a standard quality assessment tool (see Table 1).

**Table 1:** Summary of systematic reviews conducted on depression among HIV/AIDS patients included in this systematic review of reviews (*N* = 9).

**Review study**	**Geographical coverage of the review**	**Data bases covered in the search**	**Prominent tool used**	**Number of primary studies**	**Participants**	**Quality assessment**	**Main findings of the review**			**AMSTAR score**
							**Prevalence of depression symptoms**	**Prevalence of MDD**	**Associated factors**	
Bernard et al. (4)	Sub-Saharan Africa	Medline, Scopus, PsycInfo, PsycArticles, Psychology, and behavioral sciences collection African Index Medicus and African journal online	MINI for MDD & CES-D scale for depressive symptoms	16 studies for MDD& 45 papers for DS*	3,201	GRADEpro GDT tool is used for quality assessment	19% (95% CI: 18–21)	No pooled prevalence was calculated due to the variety of scales used	In PLHIV, MDD was associated with WHO clinical stage 3 or 4, poor health related quality of life, comorbidities such as tuberculosis, prior history of MDD or manic episode, female gender & advanced age	9
Amare et al. (20)	Ethiopia	MEDLINE/PubMed, PsycINFO, Google Advance Scholar, and Google Scholar	Patient Health Questionnaire-9 (PHQ-9)	13	6,649	Not assessed	36.65% (95% CI: 25.48–47.82).	Not reported	Perceived HIV stigma, Poor social support and living alone, Poor medication adherence, Clinical stage III and stage IV of HIV/AIDS, low income, and being female were among the associated factors	8
Tsai (23)	Sub-Saharan Africa	African Journals Online, African Journal Archive, Cumulative Index to Nursing & Allied Health Literature, Embase, MEDLINE, PsycINFO, and WHO African Index Medicus	PHQ-9 & CES-D	13	5,373	Quality assessment of diagnostic accuracy studies tool	29.5% (95% CI, 20.5–39.4)	13.9% (95% CI, 9.7–18.6)	Not reported	7
Necho et al. (21)	Ethiopia	PubMed, Scopus, and EMBASE		21	10,090	Modified Newcastle–Ottawa scale (NOS)	35.8% (95% CI 28.29, 43.25)	Not reported	Perceived HIV stigma, poor social support, poor medication adherence, opportunistic infection, and advanced stages of AIDS were the most common reported associated factors	10
Patel et al. (22)	Worldwide	PubMed/MEDLINE, Cochrane Review, and Scopus	PHQ-9	57	27,842	Quality assessment not done	24.4% (95% CI 12.5–42.1)	Not reported	Not reported	5
Ayano et al. (5)	East Africa	PubMed, EMBASE, SCOPUS	Diagnostic and Statistical Manual of Mental Disorders (DSM)& &PHQ_9	19	9,217	Modified version of NOS	46% (95% CI 29.30–47.54)	12.40% (4.0, 32.80)	Having opportunistic infection, perceived HIV stigma, negative life event, advanced HIV, stressful life events, hospitalization in the past 1 month, missed frequency of clinic visit, food insecurity, self-efficacy, income, frequency of follow-up, older age, urban residence and being government employee	10
DiMatteo et al. (27)	Sub-Saharan Africa	Not reported		23	11,421	Not reported	31.2%	18%	Not reported	11
Wang et al. (24)	China	EMBASE, Web of Science, PubMed, Wanfang, China Biology Medicine disc, China National Knowledge Infrastructure	Zung self-rating depression scale score in 16 studies	74	20,635	Modified version of NOS	50.8% (95% CI: 46.0–55.5%)		Not reported	11
Ciesla et al. (28)	Worldwide	Psych-Info, MEDLINE, and AIDSLINE	8 studies used DSM-III-R,	10	2,596	Not reported	Not reported	9.4%	Not reported	5

AIDS, Acquired immune deficiency syndrome; AMSTAR, Assessment of multiple systematic reviews; CS, Cross-sectional; CES-D, Center for epidemiological studies depression tool; CI, Confidence interval; DSM, Diagnostic and statistical manual of mental disorders; DiasGRADEpro GDT, Grading of recommendations assessment, development, and evaluation—guideline development tool; DS^*^, Depressive symptoms; JBI, Joanna briggs institute; HIV, Human Immune Virus; MDD, Major depressive disorder; MINI, Mini-International neuropsychiatric interview; NOS, Newcastle Ottawa quality assessment scale; PHQ-9, Patient health questionnaire-9.

#### AMSTAR Score of Included Studies

Generally, the AMSTAR score assesses 11 components of a systematic review study; was a priori design provided? Was there duplicate study selection and data extraction? Was a comprehensive literature search performed? Was the status of the publication (e.g., gray literature) used as inclusion criteria? Was a list of included and excluded studies provided? Were the characteristics of included studies provided? Was the scientific quality of included studies assessed and reported? Was the scientific quality of included studies used appropriately in formulating conclusions? Were the methods used to combine the findings of the study appropriate? Was the likelihood of publication bias assessed? and was the conflict of interest stated? Considering these parameters, the AMSTAR score of included systematic review studies are as follows: six have high-quality scores and the remaining three have a medium score (see Table 2).

**Table 2:** AMSTAR score of included reviews on depressive symptoms/major depression and associated factors on HIV/AIDS patients.

**AMSTAR criteria**	**Bernard et al. (4)**	**Amare et al. (20)**	**Tsai (23)**	**Necho et al. (21)**	**Patel et al. (22)**	**Ayano et al. (5)**	**DiMatteo et al. (27)**	**Wang et al. (24)**	**Ciesla et al. (28)**
1. Was a priori design provided?	Yes	Yes	Yes	Yes	Yes	Yes	Yes	Yes	Yes
2. Was there duplicate study selection and data extraction?	Yes	Yes	No	Yes	No	Yes	Yes	Yes	No
3. Was a comprehensive literature search performed?	Yes	Yes	Yes	Yes	Yes	Yes	Yes	Yes	Yes
4. Was status of the publication (e.g., gray literature) used as inclusion criteria?	Yes	Yes	Yes	Yes	Yes	Yes	Yes	Yes	No
5. Was a list of included and excluded studies provided?	Yes	Yes	No	Yes	No	Yes	Yes	Yes	No
6. Was the characteristics of included studies provided?	Yes	Yes	Yes	Yes	No	Yes	Yes	Yes	No
7. Was the scientific quality of included studies assessed and reported?	Yes	No	Yes	Yes	No	Yes	Yes	Yes	No
8. Was the scientific quality of included studies used appropriately in formulating conclusions?	No	No	No	No	No	No	Yes	Yes	No
9. Were the methods used to combine the findings of the study appropriate?	Yes	Yes	Yes	Yes	Yes	Yes	Yes	Yes	Yes
10. Was the likelihood of publication bias assessed?	No	No	No	Yes	No	Yes	Yes	Yes	Yes
11. Was the conflict of interest stated?	Yes	Yes	Yes	Yes	Yes	Yes	Yes	Yes	Yes

#### The Pooled Prevalence of Depressive Symptoms and Major Depressive Disorder in the Umbrella Review

**Figure 2:**
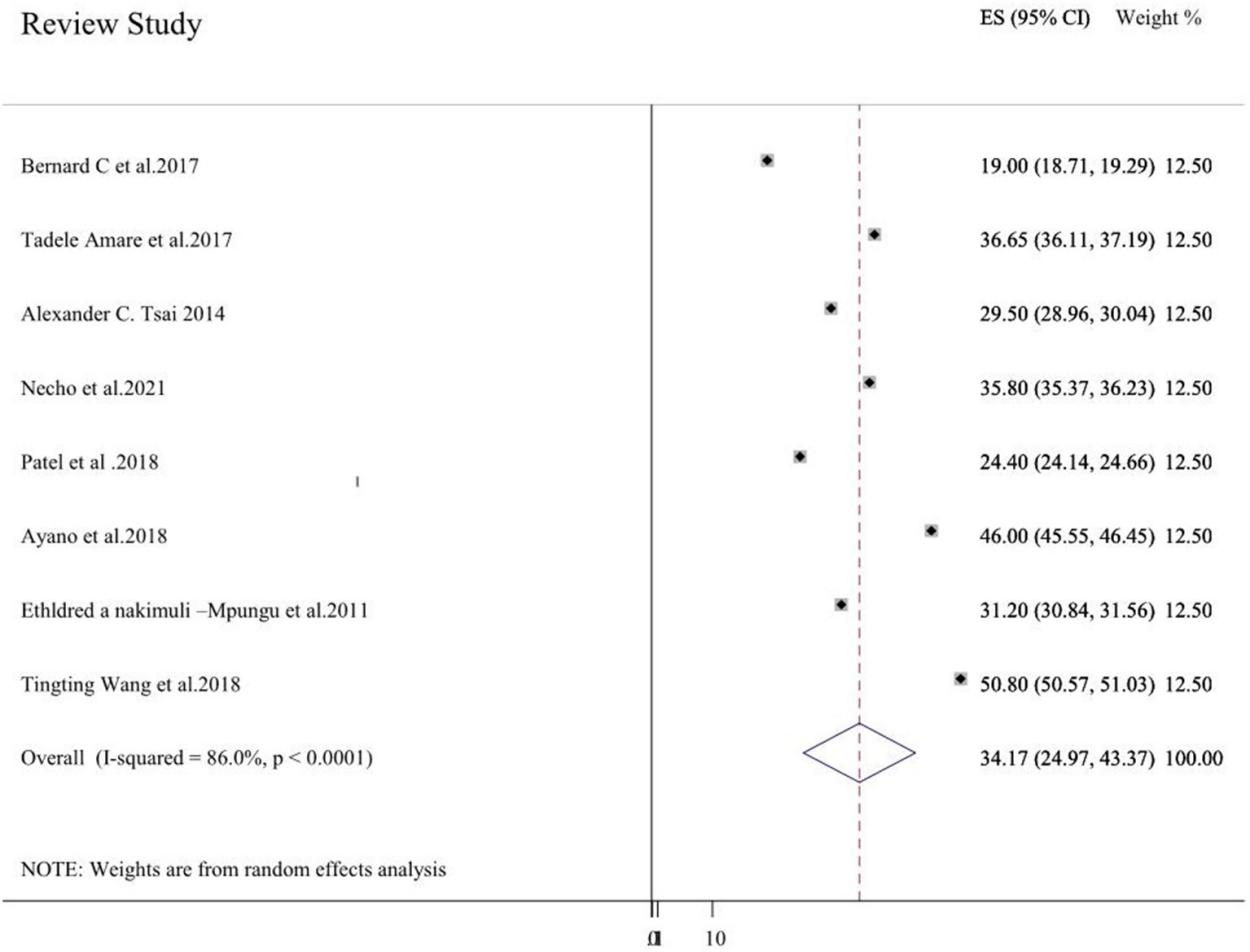
A forest plot for the systematic review of reviews on depressive symptoms.

Eight systematic review studies (3–5, 20–24) had reported depressive symptoms in HIV/AIDS patients. The prevalence of depressive symptoms among the review studies ranges from 19% in Sub-Saharan Africa (4) to 50.8% in China (24). The average depressive symptoms prevalence of included systematic review using the random effect model was 34.17% (24.97, 43.37). This average prevalence has a significant heterogeneity (*I*^2^ = 86%, *p* < 0.001) from the difference among the incorporated systematic review studies (Figure 2). Three review studies were from sub-Saharan Africa (3, 4, 23), and the average depressive symptom prevalence of these studies was 26.57% (18.01, 35.12) (*I*^2^ = 90.9%, *p* < 0.001). Two systematic review studies were from Ethiopia (20, 21) and the average depressive symptom prevalence was 36.21% (35.38, 37.04) (*I*^2^ = 82.7%, *p* < 0.001).

**Figure 3:**
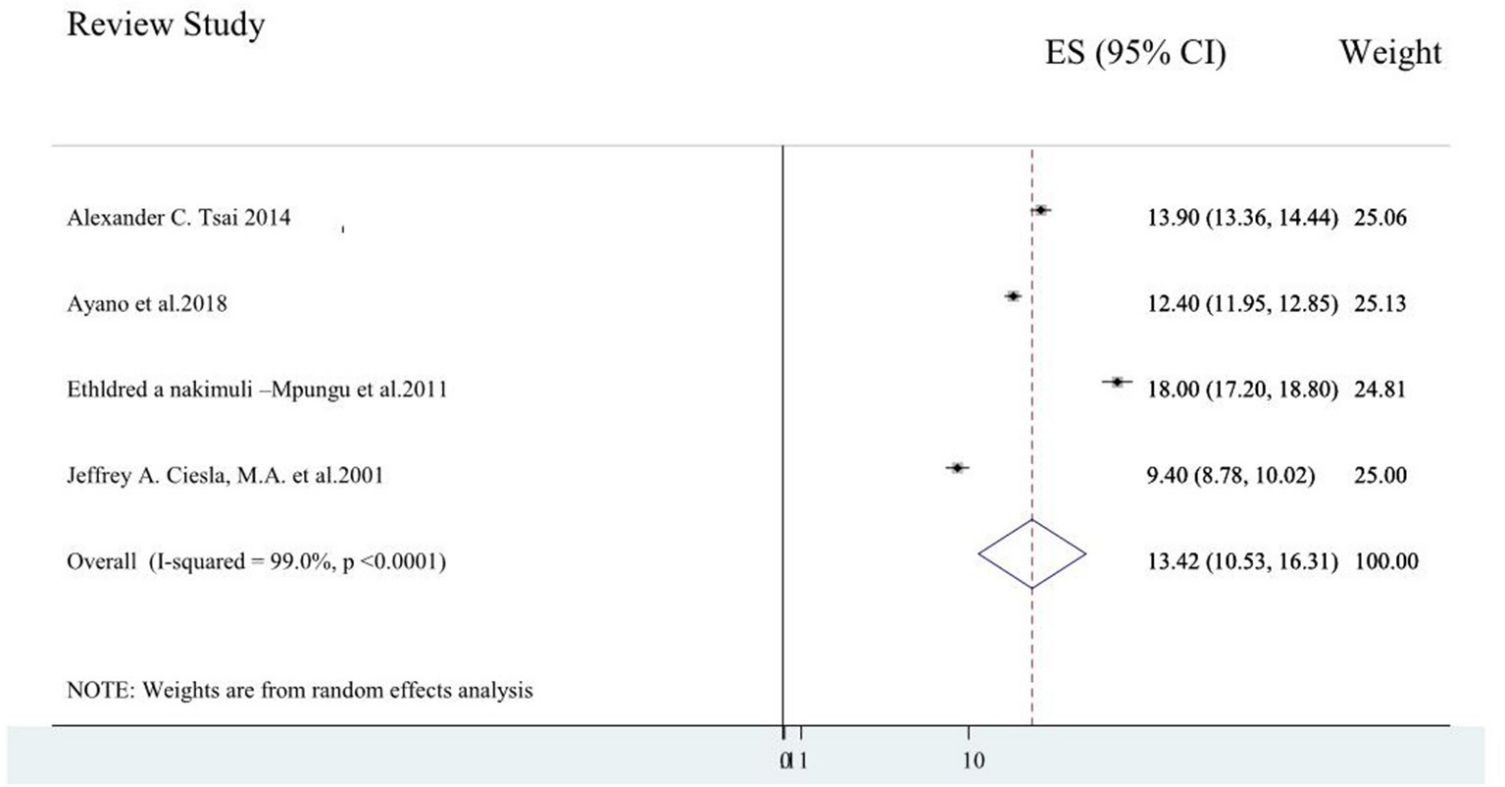
A forest plot for the systematic review of reviews on major depression.

Four systematic review studies (3, 5, 9, 23) reported data on the major depressive disorder, and the average major depression prevalence among these studies varies from 9.4% of global prevalence (9) to 18% Sub-Saharan Africa (3). The average prevalence of the major depressive disorder among the four included systematic review studies were obtained to be 13.42% (10.53, 16.31) (*I*^2^ = 90%, *p* < 0.001; Figure 3).

### Sensitivity Analysis

A one systematic review study leave-out at a time sensitivity analysis was done to explore whether a single systematic review study outweighed the average prevalence of depressive symptoms/major depressive disorder. However, the result obtained indicated that the average estimated prevalence of depressive symptoms/major depressive disorder obtained when each systematic review study is left out from pooled analysis was within the 95% confidence interval of the pooled depressive symptom/major depressive disorder prevalence. Therefore, the result of the average prevalence of depressive symptoms/ major depression in HIV/AIDS patients can be credible.

### Publication Bias of Reviews for the Pooled Prevalence of Depressive Symptoms in the Included Reviews of the Umbrella Review

**Figure 4:**
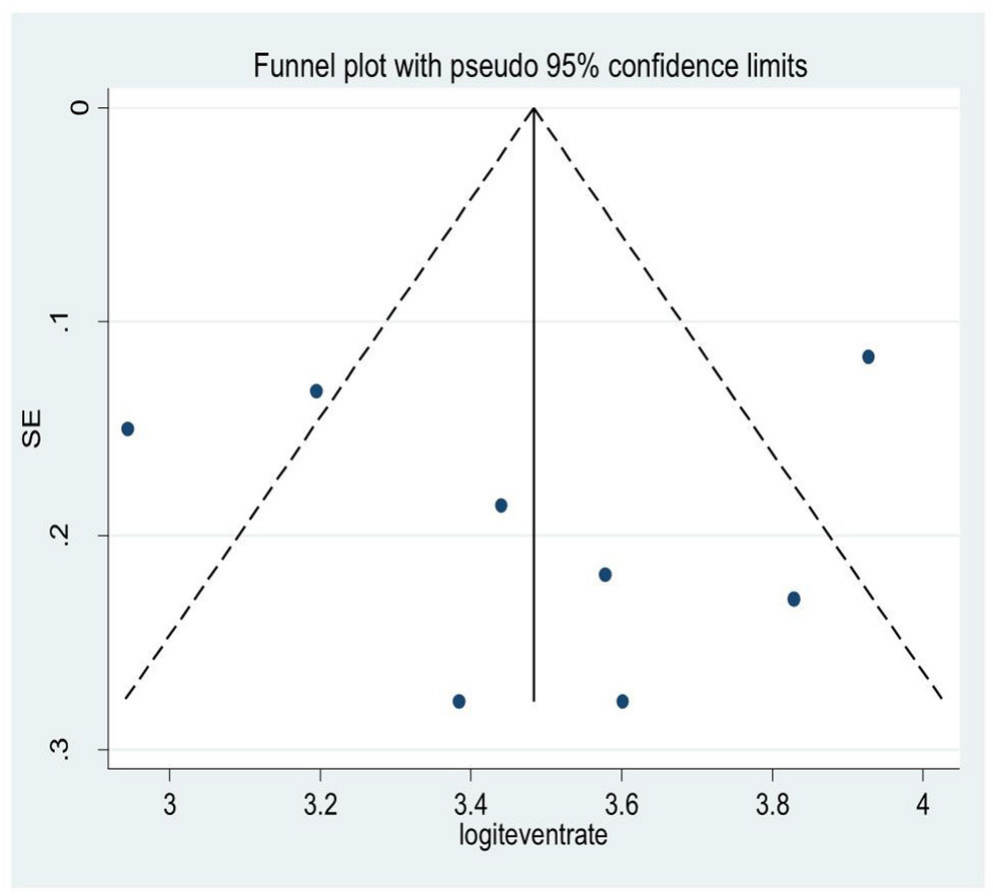
A funnel plot for the systematic review of reviews on depressive symptoms.

A snowball/visual inspection from the funnel plot for the average depressive symptom prevalence among HIV/AIDS patients showed that there exists an asymmetrical distribution of studies in the right and left margins of the funnel (Figure 4). This was further supported by the result of Egger's regression tests (*P* = 0.46). This implies there exist non-ignorable missing data during the searching or analysis process of the study.

### Association of Depressive Symptoms on Medication Non-adherence Among HIV/AIDS Patients Included in This Umbrella Review

#### Characteristics of Included Reviews

We identified six systematic review studies (2, 3, 9, 11, 25, 26) that studied the effect of depressive symptoms on antiretroviral medication non-adherence. Except for one (3) which covers sub-Saharan articles, all of the remaining included systematic review studies cover articles from the global context. A total of 442 primary studies that assessed 141,885 participants were included. PubMed/Medline and Scopus were the most searched databases by the systematic reviews (see Table 3). Three of the systematic reviews had a high-quality score on the AMSTAR quality assessment criteria and the remaining three scored medium quality (see Table 4).

**Table 3:** Summary of systematic reviews conducted on the effect of depressive symptoms on medication non-adherence among HIV/AIDS patients included in this systematic review of reviews (*N* = 6).

**Review study**	**Geographical coverage of the review**	**Data bases covered in the search**	**Number of primary studies**	**Number of participant**	**Quality assessment**	**AMSTAR score**	**Main findings of the review**
Uthman et al. (2)	Low-, Middle-, and High-Income Countries	PubMed, EMBASE and Cochrane CENTRAL	111	2,861	Not applicable	9	Achieving good ART adherence was 42% lower among those with depressive symptoms compared to those without [Pooled OR = 0.58, 95 % CI (0.55–0.62)]
DiMatteo et al. (27)	Sub-Saharan Africa	Not reported	23	11,421	Not applicable	11	Likelihood of achieving good adherence was 55% lower among those with depression symptoms compared to those without [pooled OR = 0.45 (95% CI 0.31–0.66, Tau^2^ = 0.20, *P* = 0.000)]
Sin and DiMatteo (11)	Global	PubMed and PsycINFO	29	1,293	Not applicable	7	The odds of a person adhering to ART are 83 % better if he or she is treated for depression, and the risk of non-adherence is 35% greater among those who do not receive depression treatment
Ciesla et al. (28)	Global	Psych-Info, MEDLINE, and AIDSLINE	10	2,596	Not reported	5	Depression was significantly (*P* < 0.0001) associated with non-adherence (*OR* = 0.19; 95% confidence interval = 0.14–0.25)
Springer et al. (26)	Global	PubMed, Scopus and Web of Knowledge	62	19,878	Not reported	5	Sixty-two articles examined depression, with 58% (*N* = 32/62) finding lower cART adherence and persistence. Twenty-two studies did not find a statistically significant association between depression and decreased cART adherence
Nienke Langebeek et al. (25)	Global	PubMed	207	103,836	STROBE	10	Depressive symptoms were more strongly and negatively associated with adherence to antiretroviral medications

*AMSTAR, Assessment of multiple systematic reviews; ART, Antiretroviral therapy; STROBE, Strengthening the reporting of observational studies in epidemiology; CI, Confidence interval; OR, Odds ratio*.

**Table 4:** AMSTAR score of included reviews on effect of depressive symptoms on medication non-adherence among HIV/AIDS patients included in this systematic review of reviews.

**AMSTAR criteria**	**Uthman et al. (2)**	**DiMatteo et al. (27)**	**Nancy et al. (2013)**	**Ciesla et al. (28)**	**Springer et al. (26)**	**Nienke Langebeek et al. (25)**
1. Was a priori design provided?	Yes	Yes	Yes	Yes	Yes	Yes
2. Was there duplicate study selection and data extraction?	Yes	Yes	No	No	No	Yes
3. Was a comprehensive literature search performed?	Yes	Yes	Yes	Yes	Yes	Yes
4. Was status of the publication (e.g., gray literature) used as inclusion criteria?	Yes	Yes	Yes	No	Yes	Yes
5. Was a list of included and excluded studies provided?	Yes	Yes	Yes	No	No	Yes
6. Was the characteristics of included studies provided?	Yes	Yes	Yes	No	No	Yes
7. Was the scientific quality of included studies assessed and reported?	No	Yes	No	No	No	No
8. Was the scientific quality of included studies used appropriately in formulating conclusions?	No	Yes	No	No	No	Yes
9. Were the methods used to combine the findings of the study appropriate?	Yes	Yes	Yes	Yes	Yes	Yes
10. Was the likelihood of publication bias assessed?	No	Yes	No	Yes	No	Yes
11. Was the conflict of interest stated?	Yes	Yes	Yes	Yes	Yes	Yes

#### The Pooled Odds Ratio for the Association of Depressive Symptoms on Antiretroviral Medication Adherence

All of the included systematic review studies (2, 3, 9, 11, 25, 26) reported a negative association between depressive symptoms and antiretroviral medication adherence. For example, a review study by Uthman et al. (2) reported that patients with depressive symptoms were 42 % lower to adherent for their ART than those without depressive symptoms [Pooled OR = 0.58, 95 % CI (0.55–0.62)]. Similarly, a sub-Saharan review study (3) reported that depressed patients were 55% lower adherent to their medication as compared to those without depression [pooled OR = 0.45 (95% CI 0.31–0.66, Tau^2^ = 0.20, *P* = 0.000)].

**Figure 5:**
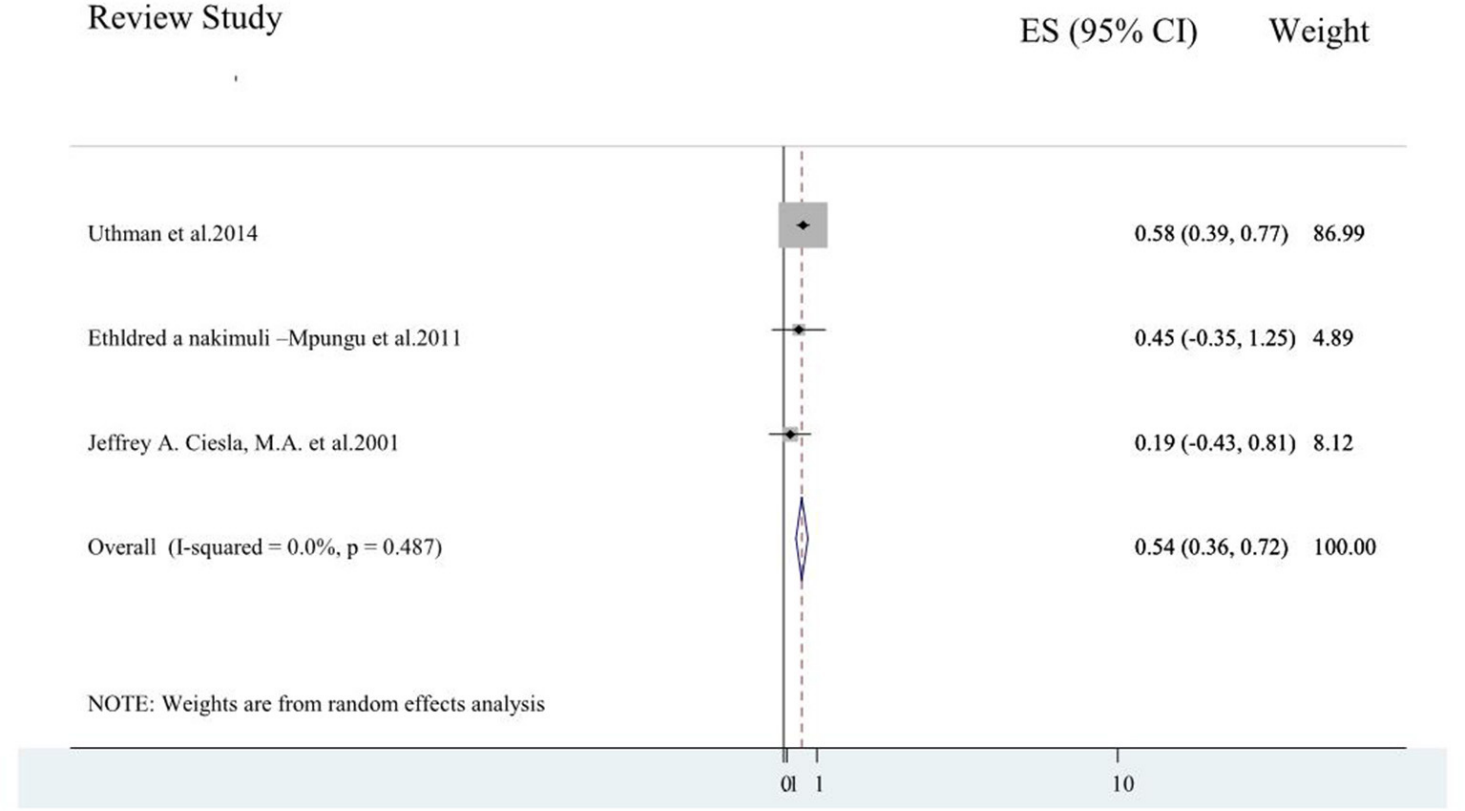
A forest plot for the systematic review of reviews of effects of depressive symptoms on antiretroviral medication adherence.

Three of the six review studies (2, 3, 9) reported the strength of association between depressive symptoms and antiretroviral medication adherence. The pooled odds ratio of adherence among patients with depressive symptoms was 0.54 (0.36, 0.72) (I-squared = 0.0%, *p* = 0.487; Figure 5).

## Discussion

Even though depressive symptoms/ major depression is highly associated with antiretroviral medication non-adherence, this has been poorly integrated into prevention policies and strategies. This could be due to the absence of concert aggregate evidence that could guide policymakers and intervention planners.

We accompanied these umbrella reviews to systematically generalize the worldwide burden of depressive symptoms/major depressive disorder and its impact on antiretroviral medication adherence.

Nine systematic review studies (3–5, 9, 20–24) that addressed 275 primary studies had reported depressive symptoms/major depression in HIV/AIDS patients. Of these systematic review studies, three assessed primary studies from sub-Saharan Africa (3, 4, 23), and two assessed primary studies from Ethiopia (20, 21). The remaining reviews cover primary studies from low and middle-income countries (22), East Africa (5), China (24), and the global context (9). The number of original studies incorporated in the systematic reviews vary from as low as 10 studies with 2,596 participants (9) to as high as 74 studies with 20,635 participants (24).

The prevalence of depressive symptoms among the review studies ranges from 19% in Sub-Saharan Africa (4) to 50.8% in China (24). The average depressive symptom prevalence among the included systematic review studies was 36.21%. This was in line with the pooled prevalence of depressive symptoms among rheumatoid arthritis patients where the pooled prevalence of depressive symptoms was 38.8% and 34.2% as measured with PHQ_9 and HADS, respectively (29). The pooled prevalence of depressive symptoms among chronic kidney patients was 39% (30) and was consistent with the current study. Additionally, this result is comparable to the average prevalence of depressive symptoms in cancer patients where 72 studies from 30 countries were pooled and resulted in 32.2% for depression symptom prevalence (31).

However, the prevalence of depressive symptoms in the present study was higher compared to the pooled estimated prevalence of depressive symptoms among general outpatient attendants (27%) (29). Moreover, the current finding was higher than the pooled prevalence of depressive symptoms among hypertensive patients (26.8%) (32). The higher rate of internalized as well as perceived stigma and socio-cultural discrimination in HIV patients could attribute to the higher magnitude of depressive symptoms (33).

On the contrary, the results of the present study were relatively lower as compared to the pooled prevalence of depressive symptoms among tuberculosis patients as reported with a systematic review and meta-analysis study of 25 studies and 4,903 participants where 45.19% of participants had depressive symptoms (34). Evidence suggests that the activation of enzymes, such as indoleamine 2, 3-dioxygenase, secondary to chronic inflammation as a result of TB infection reduces tryptophan and by this means affects serotonin production (35). Moreover, the psychosocial and health-related quality of life dysfunction that tuberculosis poses on patients would elevate the level of depressive symptoms (36, 37).

Four systematic review studies (3, 5, 9, 23) reported data on major depressive disorder, and the average major depression prevalence of these systematic review studies varies from 9.4% (9) to 18% in Sub-Saharan Africa (3). The average prevalence of the major depressive disorder among the four included systematic review studies were obtained to be 13.42%. A study on the prevalence of depression in the Community from 30 Countries between 1994 and 2014 obtained a result that was consistent with this study (38).

Moreover, six systematic reviews studies (2, 3, 9, 11, 25, 26) that included a total of 442 primary studies and 141,885 participants assessed the effect of depressive symptoms on antiretroviral medication adherence. Except for one (3) which covers sub-Saharan articles, all of these included systematic review studies cover articles from the global context. All of the included systematic review studies (2, 3, 9, 11, 25, 26) reported a negative association between depressive symptoms and antiretroviral medication adherence. For example, a review study by Uthman et al. (2) reported that patients with depressive symptoms were 42% lower adherent to their ART than those without depressive symptoms. Similarly, a sub-Saharan review study (3) reported that depressed patients were 55% lower adherent to their medication as compared to those without (pooled OR = 0.45).

Three of the six review studies reported the strength of association between depressive symptoms and antiretroviral medication adherence. The pooled odds ratio of adherence among patients with depressive symptoms was 0.54 (0.36, 0.72). This implied that people living with HIV/AIDS who had depressive symptoms were 46% less adherent to antiretroviral medication as compared to patients who had no depressive symptoms. A systematic review and meta-analysis studies reported that depressed patients had a loss of self-efficacy and the ability of decision-making capacity for treatment adherence which could attribute to this finding (39, 40).

### Strength and Limitations of This Review of Reviews

These systematic reviews of reviews have the following strengths and limitations. The primary strengths of this review of reviews are the very broad and inclusive scope of the data and the applicability of the data to the problem. This umbrella review also yields an average burden of depressive symptoms/depression and its association with treatment adherence by uniting the result of multiple systematic reviews. A separate pooled analysis was also done for depression as a condition and depressive symptoms as measured using symptom checklists. This lays pivotal evidence for policymakers and intervention planners. The constraint of the study was that this systematic review of review did not address the possible contributing factors/meta-regression was not done for depressive symptoms/major depression which had to be addressed by researchers in the future. Moreover, high heterogeneity existed for review studies included in the umbrella analysis of depressive symptoms. Furthermore, as this study did not address the impact of treatment for depressive symptoms/depression on antiretroviral medication adherence, future research and researchers should investigate this phenomenon of interest. Last but not least, due to the wider contextual scope of coverage of the umbrella review, there might be a possibility of missing eligible studies during the search process. The challenges of comparisons between different methods were also another difficulty during the analysis.

## Conclusion

This systematic review of reviews found a high pooled prevalence of depressive symptoms and major depression among people living with HIV/AIDS. The study also obtained that depressive symptoms affect patients' adherence to antiretroviral medication. These findings are very imperative as it suggests evidence for a variety of stakeholders that works for the betterment of the quality of life of people living with HIV/AIDS. The primary stakeholders are clinicians who might benefit from the early screening and treatment of depressive symptoms/disorders in people living with HIV/AIDS. Next, policymakers and intervention players should encircle depression during the development of clinical guidelines, manuals, and prevention modules for people living with HIV/AIDS.

## Data Availability Statement

The original contributions presented in the study are included in the article/supplementary material, further inquiries can be directed to the corresponding author/s.

## Author Contributions

MN premeditated the study, did the analysis and interpretation of the findings, and drafted the article. MN, BY, GA, and YZ accomplished the data search, extraction, and quality assessment of studies. YZ and MN revised the article. All authors read and approved the final draft of the manuscript.

## Conflict of Interest

The authors declare that the research was conducted in the absence of any commercial or financial relationships that could be construed as a potential conflict of interest.

## Publisher's Note

All claims expressed in this article are solely those of the authors and do not necessarily represent those of their affiliated organizations, or those of the publisher, the editors and the reviewers. Any product that may be evaluated in this article, or claim that may be made by its manufacturer, is not guaranteed or endorsed by the publisher.

## Abbreviations

AIDS: Acquired Immune Deficiency Syndrome
AMSTAR: Assessment of Multiple Systematic Reviews
ART: Antiretroviral Therapy
CS: Cross-sectional
CES-D: Center for Epidemiological Studies Depression scale
CI: Confidence Interval
DSM: Diagnostic and Statistical Manual of Mental disorders
DiasGRADEpro GDT: Grading of Recommendations Assessment, Development, and Evaluation—Guideline Development Tool
JBI: Joanna Briggs Institute
HIV: Human Immune Virus
MDD: Major Depressive Disorder
MINI: Mini-International Neuropsychiatric Interview
NOS: Newcastle Ottawa Quality Assessment Scale
PHQ-9: Patient Health Questionnaire-9
STROBE: Strengthening the Reporting of Observational Studies in Epidemiology.
